# A Bayesian Approach to Predict Football Matches with Changed Home Advantage in Spectator-Free Matches after the COVID-19 Break

**DOI:** 10.3390/e24030366

**Published:** 2022-03-04

**Authors:** Jaemin Lee, Juhuhn Kim, Hyunho Kim, Jong-Seok Lee

**Affiliations:** Department of Industrial Engineering, Sungkyunkwan University, Suwon 16419, Korea; torresss@skku.edu (J.L.); juhuhn@skku.edu (J.K.); retna319@skku.edu (H.K.)

**Keywords:** COVID-19, Bayesian hierarchical Poisson model, football, match prediction, home advantage

## Abstract

Since the coronavirus disease 2019 (COVID-19) pandemic, most professional sports events have been held without spectators. It is generally believed that home teams deprived of enthusiastic support from their home fans experience reduced benefits of playing on their home fields, thus becoming less likely to win. This study attempts to confirm if this belief is true in four major European football leagues through statistical analysis. This study proposes a Bayesian hierarchical Poisson model to estimate parameters reflecting the home advantage and the change in such advantage. These parameters are used to improve the performance of machine-learning-based prediction models for football matches played after the COVID-19 break. The study describes the statistical analysis on the impact of the COVID-19 pandemic on football match results in terms of the expected score and goal difference. It also shows that estimated parameters from the proposed model reflect the changed home advantage. Finally, the study verifies that these parameters, when included as additional features, enhance the performance of various football match prediction models. The home advantage in European football matches has changed because of the behind-closed-doors policy implemented due to the COVID-19 pandemic. Using parameters reflecting the pandemic’s impact, it is possible to predict more precise results of spectator-free matches after the COVID-19 break.

## 1. Introduction

The coronavirus disease 2019 (COVID-19) pandemic began spreading globally by the end of 2019. Sports were no exception. Four major European football leagues (Premier League, LaLiga, Serie A, and Bundesliga) went on a few months’ break after the pandemic’s outbreak, later resuming with matches played behind closed doors [1]. In the home-and-away system of professional football matches, the home team is usually believed to have a “home advantage” when playing on their ground—thanks to the enthusiastic support of a large number of home spectators—such that the home team wins relatively more at home on average. However, the home team no longer receives such enthusiastic support from home supporters because of the COVID-19 pandemic, which could reduce the benefits of existing home teams. For example, a team manager in the English Premier League once said that the opponent’s stadium is intimidating only if it is full of a crowd [2]. Experts and media frequently propose the hypothesis that the absence of spectators since the COVID-19 pandemic could affect the teams’ home advantage.

Sports match prediction is a huge business market in the modern society. Football, one of the most popular sports in the world, boasts its success because of large capital investment; the estimated market size of European football reached EUR 28.9 billion in 2019 [3]. This leads to the subsequent bloom of the neighboring industry of match prediction and gambling. The size of the global online gambling market reached USD 53.7 billion in 2019 [4]. As the market becomes more capital-intensive, sports consumers start asking for a systematic and rational decision-making process to support their investment of a large sum of money. Ever since the COVID-19 pandemic, there has been a growing need for robust match prediction models that reflect systematic changes in the COVID-19 era.

In terms of related studies, several researchers focused on identifying the presence of home advantage [5,6,7,8] and its disappearance during the COVID-19 break [9,10,11,12,13,14,15,16]. However, their findings did not consider the emerging interests in the match prediction model, that is, changes caused by the COVID-19 pandemic. Many studies have also been conducted on football match prediction using stochastic models [17], machine learning techniques [18,19,20], and domain knowledge of football [21,22]. Recently, a study attempted to predict the outcomes of football matches after the COVID-19 break [23]. Although the impact of COVID-19 was considered in this study, quantitative analyses for the changed home advantage were not performed. Unlike the previous works, we propose to quantify the changed home advantage after the COVID-19 break, and take it into account to build match result prediction models. That is, we use a Bayesian hierarchical Poisson model embedded with the COVID-19 effect to sample the right parameters for the prediction model. Next, we show an improvement in the model accuracy when using parameters sampled across multiple machine learning models.

This study assumes that professional football leagues have experienced diminished home advantage due to behind-closed-doors matches since the COVID-19 pandemic. Considering four major European football leagues, the study confirms a statistical decrease in home advantage by observing changes in the expected points and number of goals scored by the home team. With the model parameters sampled via the Bayesian framework, the study justifies its match prediction model by showing an improvement in accuracy when the systematic change in home advantage is reflected. Accordingly, this study presents to the academic society a comprehensive model reflecting the systematic changes occurring during the COVID-19 break.

## 2. Statistical Analysis

After the COVID-19 outbreak, a question has been continuously raised by the media and articles: does the home advantage decrease due to the lack of cheering from home supporters? Previous studies have performed statistical analyses on the mean, variance, and normality to determine the differences in home advantage in sports because of COVID-19 [24,25]. To verify the hypothesis of a reduced home advantage since the COVID-19 pandemic, we performed the following statistical analyses. First, we performed Welch’s unequal variances *t*-test [26] to see whether the home team’s match results changed during the COVID-19 break. Table 1 shows the *t*-test results performed on the expected points (average points a team is expected to earn on their home field) and the goal difference (goals scored by the home team minus goals scored by the away team), both averaged per league and per season. The test results show that mean values of the expected points and goal difference changed over the COVID-19 break.

**Figure 1:**
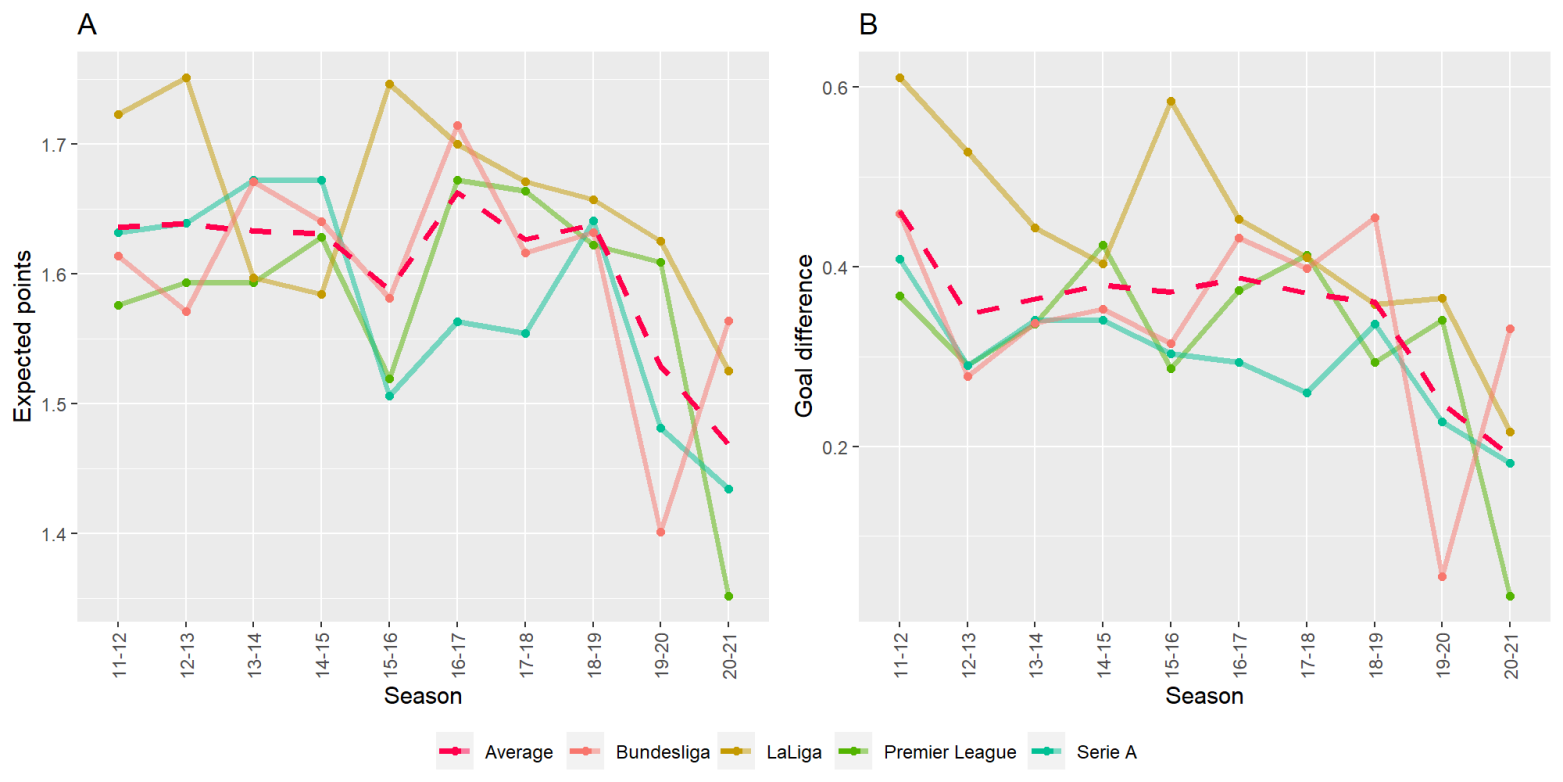
Line graphs of expected points and the goal difference for four major European football leagues. (**A**) Trend of the average expected points of the home team per league in the corresponding season. (**B**) Trend of average goal difference of the home team per league in the corresponding season. For each plot, the red dashed lines represent the average value of all four leagues per season.

Next, we visualized these quantified measures (expected points and goal difference) to determine how an unattended home match affects the match result of the home team. Figure 1 shows trends of the expected points and goal difference of the home team for each season in four major European football leagues. One can see that the expected points and the goal difference of the home team have dropped noticeably since the 2019–2020 season on average, as indicated by the red dashed line. We argue that the effect of limited spectator attendance is reflected in these two quantified measures two to three months after the COVID-19 break.

The drop in expected points is detrimental to professional football teams. The expected points for the home team were approximately 1.6 points per match before the COVID-19 break, dropping to 1.5 points after the break. Considering that every team gets to play 17 or 19 home matches per season, the drop means that each team should expect to obtain approximately two points fewer every season after the break. Further, since each league competes fiercely for one or two points for the championship or relegation zone, a difference of two points in the expected points per season has a very large impact.

We then investigated the reason for the drop in the goal difference. Regardless of how a team plays in a match, the number of goals scored is ultimately the key factor for winning or losing. Table 1 and Figure 1 show that there was a drop in goal difference over the COVID-19 break, meaning that the lack of home supporters affected the goal difference negatively. Although the (borderline) significant test results have a very large effect size (Cohen’s *d* [27]), Welch’s unequal variances *t*-test does not indicate what has caused a drop in goal difference. Therefore, we considered a hierarchy of parameters to determine hyperparameters that influence the number of goals scored by both the home and away teams in each match. Such a hierarchical model will play a major role in improving the accuracy of the football match prediction model in the post-COVID-19 era. We assumed that goals in a football match follow a Poisson distribution and considered the Bayesian hierarchical Poisson model to sample and estimate parameters in the Bayesian framework. We further proposed a Bayesian hierarchical Poisson model to predict the number of goals scored in each match.

## 3. Proposed Method

### 3.1. Bayesian Hierarchical Poisson Model

The number of goals scored in a football match follows a Poisson distribution [28]. Accordingly, Poisson regression models with various Bayesian approaches have been used to analyze football matches. There are multiple studies using a Bayesian Poisson regression model to predict football match results [15,17,29]. However, the regression model has the limitation that it is not capable of explaining serial changes in input factors. To address this issue, several studies proposed to use the Bayesian hierarchical Poisson model that can incorporate time-course factors into the model [30,31,32]. Baath also used this model to utilize team skill changes over seasons by hierarchically connecting the current team skill to that of the previous season [32]. Based on the idea that a seasonal change can be incorporated into a prediction model, this research presents a Bayesian hierarchical Poisson model with consideration of the home advantage change due to the COVID-19 break. We certainly include the other factors, such as team skill, in our prediction model.

#### 3.1.1. Model Structure

We propose a Bayesian hierarchical Poisson model to estimate the number of goals scored. The model structure is described as follows. We assume that the number of goals, ${\mathrm{GOAL}}_{\mathrm{home}}$ and ${\mathrm{GOAL}}_{\mathrm{away}}$, follow the Poisson distributions. Let *i* denote the home team, j the away team, s the season, $\lambda_{\mathrm{home},i,j,s}$ the Poisson parameter of the home team during season *s*, and $\lambda_{\mathrm{away},i,j,s}$ the Poisson parameter of the away team during season *s*. Next, the number of goals scored by the home and away teams, ${\mathrm{GOAL}}_{\mathrm{home}}$ and ${\mathrm{GOAL}}_{\mathrm{away}}$, are defined by the following Equations (1) and (2):

(1) $$\begin{array}{c} {\mathrm{GOAL}}_{\mathrm{home}}∼\mathrm{Pois}\left(\lambda_{\mathrm{home},i,j,s}\right), \end{array}$$

(2) $$\begin{array}{c} {\mathrm{GOAL}}_{\mathrm{away}}∼\mathrm{Pois}\left(\lambda_{\mathrm{away},i,j,s}\right). \end{array}$$

The Poisson parameters, $\lambda_{\mathrm{home},i,j,s}$ and $\lambda_{\mathrm{away},i,j,s}$, are defined as the product of the performance difference between the two teams and other factors.

Let ${\mathrm{SKILL}}_{i}$ denote the home team performance, ${\mathrm{SKILL}}_{j}$ the away team performance, ${\mathrm{OTHERS}}_{\mathrm{home}},s$ the other factors of the home team, and ${\mathrm{OTHERS}}_{\mathrm{away}},s$ the other factors of the away team during season s. Next, the number of goals scored by the home and away teams, $\lambda_{\mathrm{home},i,j}$ and $\lambda_{\mathrm{away},i,j}$, are defined by the following logarithmic Poisson link function Equations (3) and (4):

(3) $$\begin{array}{c} \log\left(\lambda_{\mathrm{home},i,j,s}\right)={\mathrm{OTHERS}}_{\mathrm{home},s}+{\mathrm{SKILL}}_{i,s}-{\mathrm{SKILL}}_{j,s}, \end{array}$$

(4) $$\begin{array}{c} \log\left(\lambda_{\mathrm{away},i,j,s}\right)={\mathrm{OTHERS}}_{\mathrm{away},s}-{\mathrm{SKILL}}_{i,s}+{\mathrm{SKILL}}_{j,s}. \end{array}$$

Note that parameter OTHERS is included in the Bayesian hierarchical Poisson model to capture all other external factors that influence the number of goals scored in addition to the team performance, such as the presence of supporters in the stadium. Unlike ${\mathrm{SKILL}}_{i,s}$, ${\mathrm{OTHERS}}_{\mathrm{home},s}$ and ${\mathrm{OTHERS}}_{\mathrm{away},s}$ are configured independently for each season to capture other variable factors affecting the number of goals scored.

The parameter for team performance, ${\mathrm{SKILL}}_{i,s}$, is defined as the realization of the normal distribution. Let ${\mathrm{SKILL}}_{i,s}$ denote the team performance of team *i* during season *s*. Next, the team performance in each match is defined as a sample from the team performance distribution of the last season using Equation (5).

(5) $$\begin{array}{c} {\mathrm{SKILL}}_{i,s}∼N\left({\mathrm{SKILL}}_{i,s-1},\sigma_{\mathrm{seasons}}^{2}\right). \end{array}$$

Note that parameter ${\mathrm{SKILL}}_{i,s}$ follows a normal distribution with its mean set as the team performance of the previous season, ${\mathrm{SKILL}}_{i,s-1}$, and its standard deviation is set to $\sigma_{\mathrm{seasons}}$. Naturally, the team performance varies by season due to events in the team roster, such as transfers and injuries, but the change is usually not drastic. Thus, the model assumes that the team performance of a season is affected by the previous season, with equal variation throughout all seasons.

#### 3.1.2. Model Fitting

We used an open-source Bayesian analysis framework program, JAGS [33] (version 4.3.0), to fit the prediction model above. Specifically, we used RJAGS [34] (version 4.10), which was implemented using the statistical analysis program R [35] (version 4.0.5). The program performs Gibbs sampling [36] to estimate parameters of the aforementioned Bayesian hierarchical Poisson model [32]. To prevent prior knowledge from entering the model parameter estimation, the prior distributions of the parameters are set to relatively non-informative priors (normal or uniform) according to Equations (6)–(10):

(6) $$\begin{array}{c} \mathrm{OTHERS}∼N(0,4^{2}), \end{array}$$

(7) $$\begin{array}{c} {\mathrm{SKILL}}_{i,1\mathrm{st}\_\mathrm{season}}∼N\left(\mu_{\mathrm{teams}},\sigma_{\mathrm{teams}}^{2}\right), \end{array}$$

(8) $$\begin{array}{c} \mu_{\mathrm{teams}}∼N\left(0,4^{2}\right), \end{array}$$

(9) $$\begin{array}{c} \sigma_{\mathrm{teams}}∼U\left(0,3\right), \end{array}$$

(10) $$\begin{array}{c} \sigma_{\mathrm{seasons}}∼U\left(0,3\right). \end{array}$$

Given that the average number of goals per match by a team is fewer than two in European football leagues [37], we believe that this setting is non-informative enough for our study.

### 3.2. Home Advantage

Home advantage is the value of interest in this research, and shows how the external factors of home and away affect the match result. Accordingly, we define ${\mathrm{HA}}_{s}$ as the home advantage of a home team against an away team during season *s*. We calculate ${\mathrm{HA}}_{s}$ based on primarily sampled parameters (*SKILL* and OTHERS), assuming that the home advantage is commonly shared among teams within the same league. Mathematically, home advantage can be understood as the difference in other external factors between the home and away teams. As the situation changes over time, external factors can differ by season. Thus, parameter ${\mathrm{HA}}_{s}$ can be quantified as the home advantage in a particular season *s* using Equation (11).

(11) $$\begin{array}{c} {\mathrm{HA}}_{s}=\exp\left({\mathrm{OTHERS}}_{\mathrm{home},s}\right)-\exp\left({\mathrm{OTHERS}}_{\mathrm{away},s}\right) \end{array}$$

We can notice from Equations (3) and (4) that Equation (11) holds under the assumption that home and away teams have the same ability. Therefore, ${\mathrm{HA}}_{s}$ is determined by the difference between the Poisson parameters, as shown in Equation (12).

(12) $$\begin{array}{c} {\mathrm{HA}}_{s}=\lambda_{\mathrm{home},s}-\lambda_{\mathrm{away},s} \end{array}$$

In addition, the difference in the average number of goals scored varies depending on whether it is a home or away match. This value quantifies the home advantage that varies seasonally due to COVID-19 and other factors.

### 3.3. Additional Features for Prediction

We propose a football match prediction model using the parameters obtained from Bayesian hierarchical Poisson model-like features. In the existing football match prediction machine learning models, the statistics (e.g., win, draw, loss, point, goals for, goals against) are used as features. Such cumulative statistics provide only a limited amount of information about the difference between team performance and the change in match pattern since the COVID-19 pandemic. Subsequently, it is very hard to obtain a high accuracy of match prediction. Therefore, we want to improve the match prediction accuracy of machine learning models by using the home advantage and each team’s SKILL parameter as additional features obtained from the proposed model. Section 4.4 describes the advantages of this approach.

## 4. Experiments

In this section, we visualize the distribution of parameters (e.g., SKILL and HA) obtained from the aforementioned Bayesian hierarchical Poisson model, and we use these parameters to show score prediction and match prediction results. We also use the parameters we obtain as additional features for machine learning models in football match prediction to see how their performance can be improved.

### 4.1. Dataset

We used league data of four major European football leagues (Premier League, LaLiga, Serie A, and Bundesliga) for the most recent seasons (2011–2012 to 2020–2021). Notice that we consider ‘home’ and ’away’ only for each of the features, and exclude ’neutral venue’. A neutral venue is rarely used to play a game in the European football leagues [38], which means that the amount of data for ’neutral’ was not enough to estimate the parameters. All the data are used for visualization in Section 4.2. In contrast, we divided the data into training and test sets for the post-match results prediction models in Section 4.3 and Section 4.4. The training set comprised nine seasons before the COVID-19 break, and the test set consisted of the 2020–2021 season, when most matches were played behind closed doors due to the COVID-19 pandemic. Parameters SKILL and HA after the COVID-19 break were sampled with 400 matches of the 2019–2020 season after the COVID-19 break, and so information of the test sets is not included in the post-match prediction model.

### 4.2. Visualization of Parameters

Using the Bayesian hierarchical Poisson model presented in Section 3, we sampled SKILL per season and team and sampled HA for seasons before and after COVID-19 breaks. A total of 100,000 samples were sampled, each using Markov chain Monte Carlo [39,40] sampling, of which, the first 10,000 samples were burned in.

**Figure 2:**
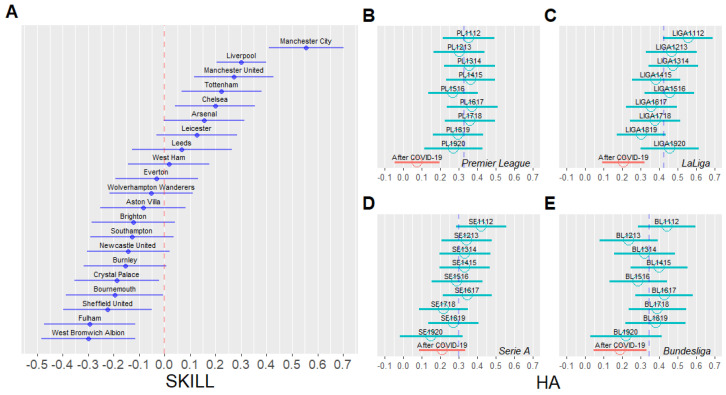
Parameters sampled from the proposed model in Section 3. (**A**) Caterpillar plot of the SKILL parameter per team in the Premier League for matches after the COVID-19 break. We adjusted the SKILL parameter such that the average is zero because SKILL is the relative parameter in the same league. The line length of the caterpillar plot represents a 95% credible interval. (**B**–**E**) Caterpillar plot of the home advantage (HA) of four major European football leagues in the 10 most recent seasons, namely B for English Premier League, C for Spanish LaLiga, D for Italian Serie A, and E for German Bundesliga. “After COVID-19” represents the 2019–2020 and 2020–2021 season matches since the leagues were suspended because of COVID-19 in March 2020. The blue dashed line represents the average HA for the 10 most recent seasons before the COVID-19 break. The line length of the caterpillar plot represents a 95% credible interval.

Figure 2A represents the distribution of SKILL in the English Premier League after the COVID-19 break: the larger the SKILL parameter, the more to the right it appears. Parameter SKILL is an indicator of a team’s ability to control goals scored/conceded and can be understood as that team’s performance. For instance, the most powerful team in the English Premier League after the COVID-19 break is Manchester City.

Figure 2B is a visualization of the distribution of HA, as defined in Section 3. Here, “After COVID-19” represents the collection of matches after the hiatuses of four major European leagues in March 2020 due to the COVID-19 pandemic. In all four leagues, the mean values of HA in matches after the COVID-19 break were lower than those of the other nine seasons before the COVID-19 break.

The mean value of HA in all matches in four European leagues from the 2011–2012 season to the season immediately before the COVID-19 break was 0.35. The mean value of HA after the COVID-19 break was 0.17. We observed that HA after the COVID-19 break was 0.18, lower on average compared with the other seasons before the COVID-19 break. In other words, the number of goals scored by the home team decreased by an average of 0.18 goals, while considering the performance gap between the teams. This shows that COVID-19 negatively affected the home advantage for all four major European football leagues.

### 4.3. Score Prediction by Sampling

We used the parameters obtained from the model in Section 3 to simulate and predict match results. As mentioned in Section 4.1, more than 400 match results of the 2019–2020 season after the COVID-19 break were analyzed to estimate the distributions of OTHERS and SKILL after the COVID-19 break. Next, we calculated Poisson parameter λs in Equations (3) and (4) using the estimated parameters of OTHERS and SKILL. With the calculated λ, pairwise goal distributions of the home and away teams are reconstructed for the matches after the COVID-19 break. In addition, the home goals and away goals were simulated by pairwise sampling from each goal distribution. As a result of the simulation, we obtained the simulated distribution of the predicted scores of a football match.

**Figure 3:**
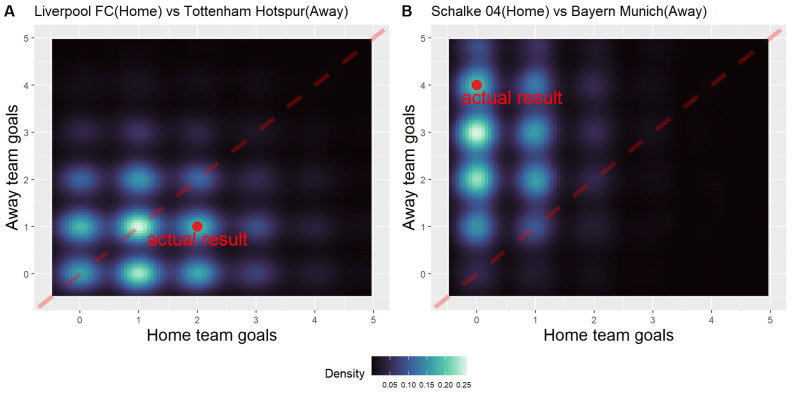
Distribution of simulated score. The brighter the point, the more frequent the score results are. The red dashed line represents the set of tie matches. The location of the red dot shows the actual match results between the two teams during the 2020–2021 season. (**A**) Liverpool FC vs. Tottenham Hotspur. (**B**) Schalke 04 vs. Bayern Munich.

The following shows two exemplary match result predictions during the 2020–2021 season since COVID-19: (1) Liverpool FC vs. Tottenham Hotspur in the English Premier League and (2) Schalke 04 vs. Bayern Munich in the German Bundesliga. We estimated SKILL parameters for Liverpool FC, Tottenham Hotspur, Schalke 04, and Bayern Munich, along with the OTHERS parameters in the Premier League and Bundesliga from the period after the COVID-19 break. Using these two parameters, we calculated $\lambda_{\mathrm{home}}$ and $\lambda_{\mathrm{away}}$ for each match. Parameter values for the two exemplary matches are listed in Table 2. It is possible to simulate the match result based on sampling from a Poisson distribution with $\lambda_{\mathrm{home}}$ and $\lambda_{\mathrm{away}}$. Figure 3 shows the distribution of scores for two exemplary matches, simulated 10,000 times. If the score distribution of a team is greater than that of the other team, the distribution becomes skewed to one side. If two teams are more likely to draw, the scores are distributed diagonally between them. In addition, the most frequently generated score was identified through pairwise score sampling. Furthermore, it is possible to calculate the probability of winning or losing using several simulation results and predict the match results. Table 2 compares the actual match result of the 2020–2021 season with our simulated results of the most frequent score, win rate, draw rate, and loss rate.

### 4.4. Match Prediction Model with Additional Features

In the previous subsections, we used the Bayesian hierarchical Poisson model to estimate the change in the HA and SKILL parameters in the matches after the COVID-19 break. As mentioned in Section 3.3, we then attempted to improve the prediction performances of machine learning models using the estimated parameters as additional input features. Three sets of input features were prepared to validate the effect of the additional features. The first set, as a baseline, does not include the OTHERS, HA, and SKILL parameters but contains the match outcomes, such as the number of goals. In addition to the features in the first set, the second set includes the OTHERS, HA, and SKILL parameters. However, the parameters do not consider the effect of the COVID-19 break. The third set not only includes the parameters but also considers their changes due to the COVID-19 break. The feature sets are described below in more detail.

**Feature set 1:** The set contains only cumulative match outcomes from previous seasons as input features. Its composition is as follows:

- Win: Number of matches won in the last season;
- Draw: Number of matches drawn in the last season;
- Loss: Number of matches lost in the last season;
- Goals_scored: Number of goals scored in the last season;
- Goals_conceded: Number of goals conceded in the last season;
- Points: Final points in the last season;
- Promoted: Recently promoted to the league in the last season.

**Feature set 2:** The set contains the seven features in feature set 1 and the OTHERS, HA, and SKILL parameters estimated from the Bayesian hierarchical Poisson model. However, ${\mathrm{OTHERS}}_{\mathrm{home}}$ and ${\mathrm{OTHERS}}_{\mathrm{away}}$ were fixed at a single seasonal value such that HA would not change after the COVID-19 break. Therefore, they have limited information on the changed home advantage due to the COVID-19 break. The additional features are as follows:

- *OTHERS*(fixed): Mean of sampled *OTHERS* parameters from the uniform *HA* model;
- *SKILL*(fixed): Mean of sampled *SKILL* parameters from the uniform *HA* model;
- *HA*(fixed): *HA* value from the uniform *HA* model.

**Feature set 3:** The set contains the seven features in feature set 1 and the OTHERS, HA, and SKILL parameters estimated from the Bayesian hierarchical Poisson model. The additional features are not fixed but variable at each season so that their changes due to the COVID-19 break can be utilized in the prediction models. We believe that, among the three sets, this set is the most suitable for match result prediction because it contains the information about the changed home advantage, if it exists. The additional features are as follows:

- *OTHERS*: Mean of sampled *OTHERS* parameters from the changed *HA* model;
- *SKILL*: Mean of sampled *SKILL* parameters from the changed *HA* model;
- *HA*: *HA* value from the changed *HA* model.

The objective of the classification models in the experiment was to predict the result of the next season’s showdown with pairwise features of the two opposing teams’ previous seasons. Evaluation metrics for matching the prediction results of four major European football leagues in the 2020–2021 season are the prediction accuracy of match results (win, draw, or loss) and *ranked probability score* (RPS) [41]. RPS is a measure of how well probability distributions are predicted when matching the actual outcomes. Therefore, several studies employed this measure to evaluate football match outcomes [42,43]. The RPS metric is defined by

(13) $$\begin{array}{c} \mathrm{RPS}=\frac{1}{r-1}\sum_{i=1}^{r}{\left(\sum_{j=1}^{i}\left(p_{j}-e_{j}\right)\right)}^{2}, \end{array}$$

where r is the number of outcomes (in this case, r=3: win, draw, or loss), $p_{j}$ is the predicted probability of outcome j, and $e_{j}$ is the actual probability of outcome j. Due to the fact that a smaller value implies how close the distribution is to the observed value, it indicates a better outcome. The actual probabilities of a real match are expressed as 0 and 1. If an actual match result is a win for the home team, the actual probability e is (1, 0, 0). We used the RPS averaged for all predicted matches, ${\mathrm{RPS}}_{\mathrm{avg}}$, computed as follows:

(14) $$\begin{array}{c} {\mathrm{RPS}}_{\mathrm{avg}}=\frac{1}{n}\sum_{k=1}^{n}{\mathrm{RPS}}_{k}, \end{array}$$

where n is the number of predicted matches.

Using the two evaluation metrics shown above, we compared the performance of various machine learning models to confirm the performance improvement of additive features. We chose several widely used classification models in machine learning: logistic regression, multilayer perception, random forest, linear support vector machine, and naïve Bayes. In addition, we proposed a classifier, *score sampling*, as a method to predict match results using the most frequent score among the simulations in Section 4.3. Note that the hyperparameters of the classifiers in the experiment are set as the values displaying the highest cross-validation accuracy on average for all three feature sets. The prior of naïve Bayes is the same as the distribution of match results (win, draw, or loss) in the training set.

Table 3 shows the hyperparameters and the experiment results of classifiers. The bold-faced result indicates the best performance among the three feature sets of a classifier. When comparing feature set 1 against feature sets 2 and 3, there is a clear improvement in the prediction performance. This shows that the prediction performance depends on the key information from additional features obtained by sampling. Compared with feature set 2, the accuracy improves when using feature set 3 in five out of six classifiers. Moreover, ${\mathrm{RPS}}_{\mathrm{avg}}$ generally improves using feature set 3 for various models, implying that these additional features facilitate a better prediction of not only the exact match results (win, draw, or loss) but also the distribution of match results. Among the aforementioned models, the model with the best prediction accuracy for matches after the COVID-19 break is *score sampling* using the features with additional information retrieved from matches after the COVID-19 break. As a result, we confirmed that it is the most suitable to predict a football match after the COVID-19 break using features that reflect the changed home advantage and team performance after the COVID-19 break.

## 5. Conclusions

This research began as a way of answering the following question: “How did the behind-closed-doors matches due to the COVID-19 pandemic affect the home team’s chance of winning?” We identified the difference in the expected points and number of goals scored by the home team between the seasons before and after the COVID-19 break using a two-sample *t*-test. The test results show that the expected points and, most importantly, the goal difference decreased over the COVID-19 break. Accordingly, this study introduced a Bayesian hierarchical Poisson model to estimate the home advantage and teams’ performance hierarchically as one of the hyperparameters that influence the number of goals scored. It found that the estimated home advantage of four major European football leagues (Premier League, LaLiga, Serie A, and Bundesliga) diminished after the COVID-19 break. The research also predicted the parameter that influences the number of goals scored by both the home and away teams using the Poisson distribution of goals with estimated parameters and predicted the score of each match result through a sampling-based simulation. In addition, the research confirmed that the accuracy of the prediction model was improved when the sampled parameters, SKILL, OTHERS, and HA, were included as the additional features in various machine learning models for football match prediction. Moreover, this study empirically identified the change in home advantage because of the COVID-19 pandemic. Nonetheless, we can barely say that our methods are sufficiently accurate for football match prediction because their test accuracy values were slightly greater than 0.5, as they were in other studies. Considering that a football game is subject to high uncertainty, it is an intrinsic limitation of football match prediction. To overcome the limitation, our future research direction is to further refine the parameters of the Bayesian hierarchical Poisson model by considering more features and utilizing diverse domain knowledge from football games. We believe that, in this way of making unmeasurable information tangible, we can design a more accurate match prediction model.

## Figures and Tables

**Table 1:** Welch’s unequal variances *t*-test on expected points and goal difference.

Test Statistic	Expected Points	Goal Difference
*t*	2.3451	2.3049
df	7.6454	7.7808
*p*-value	0.0485	0.0510
95% confidence interval	[0.0011, 0.2730]	[−0.0009, 0.3371]
mean_before_COVID-19	1.6214	0.3671
mean_after_COVID-19	1.4843	0.1990
effect size (Cohen’s *d*)	−1.4612	−1.3731

**Table 2:** Score prediction results of the exemplary matches.

	Home/Away	Team Name	Parameters			Simulated Results			Most Frequent Score	Actual Outcome
			Mean_*SKILL*	Mean_*OTHERS*	λ	Win	Draw	Loss		
Match1	Home	Liverpool FC	0.301	0.272	1.42	0.425	0.273	0.302	1	2
	Away	Tottenham Hotspur	0.224	0.212	1.14	0.302	0.273	0.425	1	1
Match2	Home	Schalke 04	−0.314	0.432	0.64	0.039	0.091	0.870	0	0
	Away	Bayern Munich	0.561	0.300	3.23	0.870	0.091	0.039	3	4

**Table 3:** Result of various match prediction models.

Classifier	Feature Set 1		Feature Set 2		Feature Set 3		Hyperparameter
	Test Accuracy	${\mathrm{RPS}}_{\mathrm{avg}}$	Test Accuracy	${\mathrm{RPS}}_{\mathrm{avg}}$	Test Accuracy	${\mathrm{RPS}}_{\mathrm{avg}}$	
**Logistic regression**	0.5062	0.2011	0.5208	0.2008	**0.5229**	**0.1999**	C = 10 (L2 regularization)
**MLP**	0.5076	**0.2003**	0.5145	0.2010	**0.5186**	0.2009	hidden layer = 2, hidden node = (3, 3)
**Random forest**	0.4695	0.2123	0.4889	0.2100	**0.5020**	**0.2073**	max features = 5, n tree = 100
**Linear SVM**	0.4951	0.2050	0.5159	0.2023	**0.5193**	**0.2015**	C = 1 (L2 regularization)
**Naïve Bayes**	0.4792	**0.1165**	**0.4819**	0.1175	0.4778	0.1177	prior = (0.3, 0.24, 0.46)
**Score sampling**	N/A	N/A	0.5214	**0.2997**	**0.5249**	0.2998	simulated 10,000 times
**Average** **(except score sampling)**	0.4915	0.1870	0.5044	0.1863	**0.5081**	**0.1855**	

## Data Availability

The data underlying the results presented in the study are available from https://www.whoscored.com (accessed on 2 March 2022). The authors had no special access privileges to the data.

## Abbreviations

The following abbreviations are used in this manuscript:

COVID-19	coronavirus disease 2019
HA	home advantage
MLP	multilayer perception
SVM	support vector machine
